# Author’s reply to comment on: A higher frequency of physical activity is associated with reduced rates of SARS-CoV-2 infection

**DOI:** 10.1080/13814788.2023.2188015

**Published:** 2023-03-21

**Authors:** Ilan Green, Eugene Merzon, Shlomo Vinker, Avivit Golan-Cohen, Ariel Israel, Mickey Scheinowitz, Reuven Ishai, Eli Magen, Shai Ashkenazi

**Affiliations:** a Leumit Health Services, Tel Aviv, Israel; b Department of Family Medicine, Sackler Faculty of Medicine, Tel Aviv University, Tel Aviv, Israel; c The Adelson School of Medicine, Ariel University, Ariel, Israel; d Department of Biomedical Engineering, Faculty of Engineering, Tel-Aviv University, Tel-Aviv, Israel; e Sylvan Adams Sports Institute, School of Public Health, Sackler Faculty of Medicine, Tel-Aviv University, Tel-Aviv, Israel; f Neufeld Cardiac Research Institute, Sheba Medical Center, Tel-Hashomer, Israel; g Department of Ear, Nose and Throat, and Head and Surgery, Rambam Medical Center, Haifa, Israel; h Faculty of Medicine, Technion Israel Institute of Technology, Haifa, Israel; i Clinical Immunology and Allergy Division, Medicine C Department, Barzilai University Medical Center, Ben-Gurion University of the Negev, Ashkelon, Israel

We thank for the opportunity to respond to the comments by Funakoshi et al. on our article ‘A higher frequency of physical activity is associated with reduced rates of SARS-CoV-2 infection,’ published in the European Journal of General Practice [1,2]. We appreciate the insights and suggestions regarding the study design and data reliability.

Regarding measuring physical activity, we agree that using validated questionnaires, such as the international physical activity (PA) questionnaire (IPAQ), can provide more accurate measures of physical activity. However, we conducted a retrospective study, utilising the information from electronic medical records (EMR) database with all limitations of this study design. Unfortunately, use of the validated questionnaire is impossible in the routine HMO practice; thus this data is not collected in the EMR database.

Regarding selecting adjustment variables in the multivariable model, we acknowledge that including COVID-19 protection acts, such as hand hygiene, wearing masks, and stay-at-home measures, in the analysis, may provide additional insights. However, unfortunately, this vital information is not collected in the EMR database. High rates of SARS-CoV-2 infection may be related to increased person-to-person contact and poor hygiene driven by behavioural patterns related to chronic disorders (ADHD, uncontrolled diabetes) [3,4], which were included in the multivariable analysis.

Regarding the excluded population, we understand that providing the numbers and reasons for exclusion can give readers a better understanding of the study population. In our study, we included only patients aged 18 years or older for whom physical activity information was collected and who underwent at least one RT-PCR test for SARS-CoV-2. From this cohort, we excluded patients who were not eligible for physical activity, such as those with congestive heart failure, dementia, hepatic, lung and renal diseases, and cancer. This allowed us to focus on a population that could engage in physical activity and obtain more precise results regarding the association between physical activity and SARS-CoV-2 infection rates. The total amount of excluded subjects was relatively low.

Again, we appreciate the comments and suggestions, and we will consider them in our future studies.
